# Data on microbiological quality assessment of rural drinking water supplies in Poldasht county

**DOI:** 10.1016/j.dib.2018.02.003

**Published:** 2018-02-07

**Authors:** Mahmood Yousefi, Hossein Najafi Saleh, Mehdi Yaseri, Amir Hossein Mahvi, Hamed Soleimani, Zhyar Saeedi, Sara Zohdi, Ali Akbar Mohammadi

**Affiliations:** a Department of Environmental Health Engineering, School of Public Health, Tehran University of Medical Sciences, Tehran, Iran; b Torbat Heydariyeh University of Medical Sciences, Torbat Heydariyeh, Iran; c Department of Epidemiology and Biostatistics, School of Public Health, Tehran University of Medical Sciences, Tehran, Iran; d Center for Solid Waste Research, Institute for Environmental Research, Tehran University of Medical Sciences, Tehran, Iran; e Students Research Committee, Neyshabur University of Medical Sciences, Neyshabur, Iran; f Department of Environmental Health Engineering, Neyshabur University of Medical Sciences, Neyshabur, Iran

**Keywords:** Drinking water, Fecal coliform, Residual chlorine, Turbidity, Village of Plodasht

## Abstract

In this research, the villages with water supply systems under the supervision of the Water and Wastewater Company in Poldasht County, Iran in 2015 was studied. 648 samples were taken from 57 villages during 12month period to test for microbial quality according to the latest guidelines of WHO. Fecal coliform, coliform, turbidity, pH and free residual chlorine were analyzed. Also we used linear Regression statistical analysis for collected data. Result of Data showed that 13.6% of the villages under study had contaminated water resources. In 100 percent of the water sample resource the turbidity level was less than Iranian maximum permissible levels (5 NTU). There was a linear relation between the Free residual color and Coliform in different month of follow up (*r* = −0.154, *P* < 0.001). Data suggests water resources should be comprehensively planned and monitored keeping in view the WHO recommended parameters.

**Specifications table**

**Table:** 

Subject area	Water microbiology
More specific subject area	Index microbial
Type of data	Tables, Figures
How data was acquired	All the examinations were done according to the 21st edition of Standard Methods for the Examination of Water and Wastewater in 2005.
Data format	Raw, Analyzed
Experimental factors	Free residual chlorine and pH were measured by a colorimeter kit on an annually basis. Most Probable Number (MPN) was done on nine tube cultivation basis. And also Turbidity test was done using HACH turbidity meter.
Experimental features	Determine the content levels of Index microbial
Data source location	Poldasht, West Azerbaijan province, Iran
Data accessibility	Data are accessibility in this article

**Value of data**

• The contamination of water with fecal coliform, turbidity increased risk of water borne disease to humans who use those waters.
• The presence of coliform bacteria in drinking water has been connection, inability to maintain a disinfectant residual in the water distribution system.
• It is necessary to monitor carefully chlorination practice in drinking water distribution system of rural area of Poldasht city and to ensure that the residual chlorine is available at consumer end.

## 1 Data

**Fig. 1:**
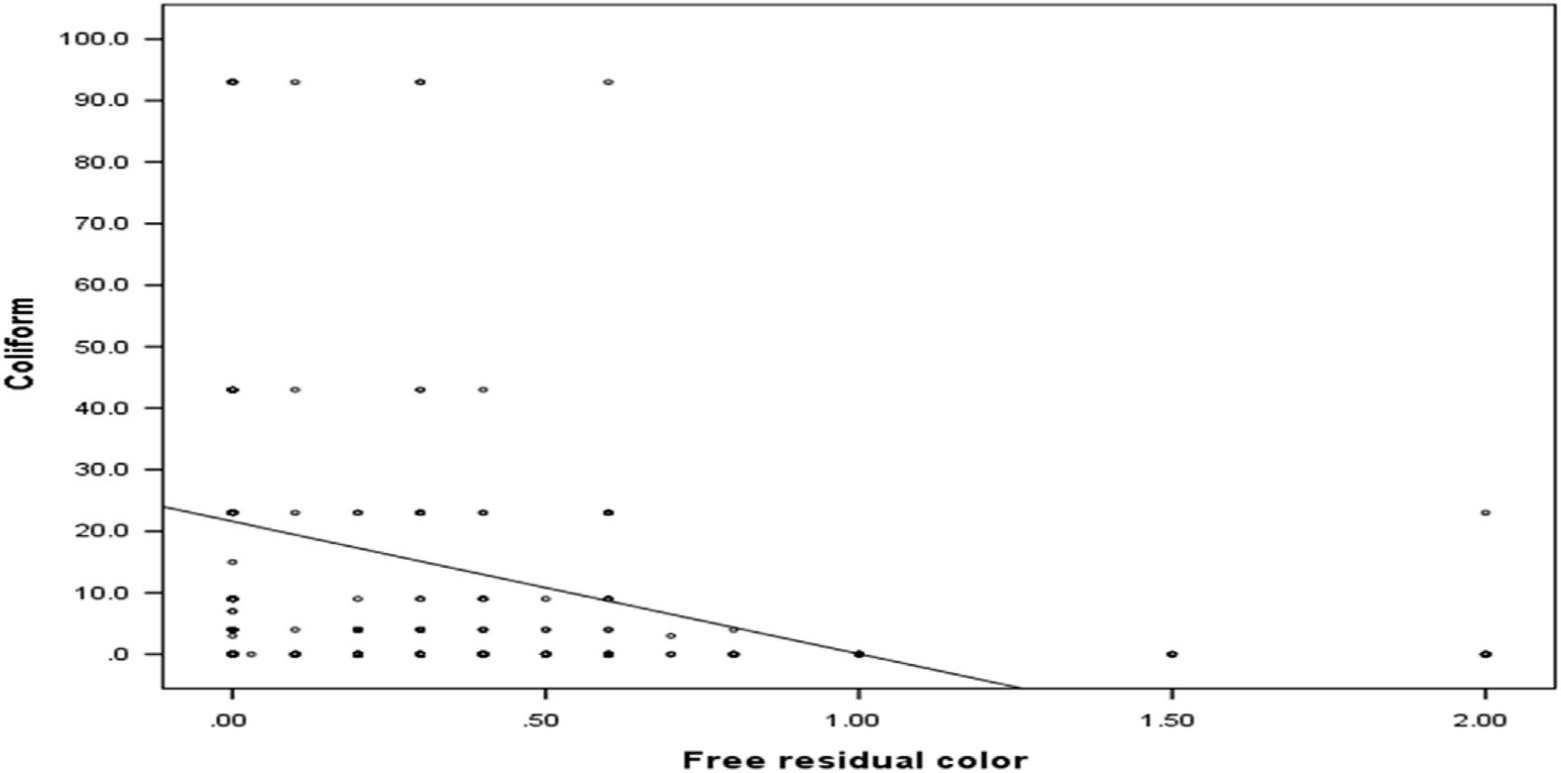
Relationship between Free chloration residual and Coliform in the different months in the drinking water of Poldasht's villages.

**Fig. 2:**
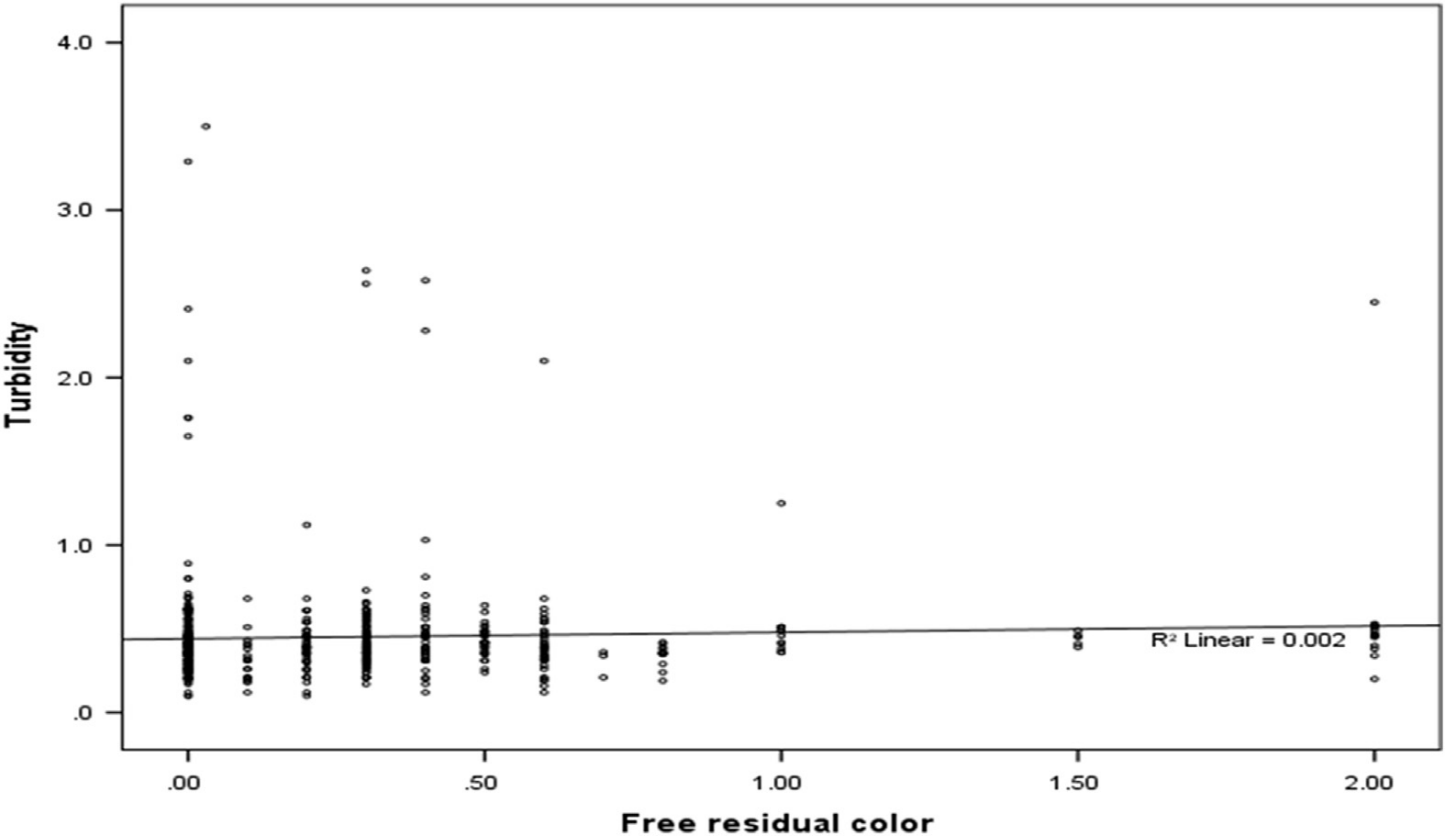
Relationship between Free chloration residual and Turbidity in the different months in the drinking water of Poldasht's villages.

**Fig. 3:**
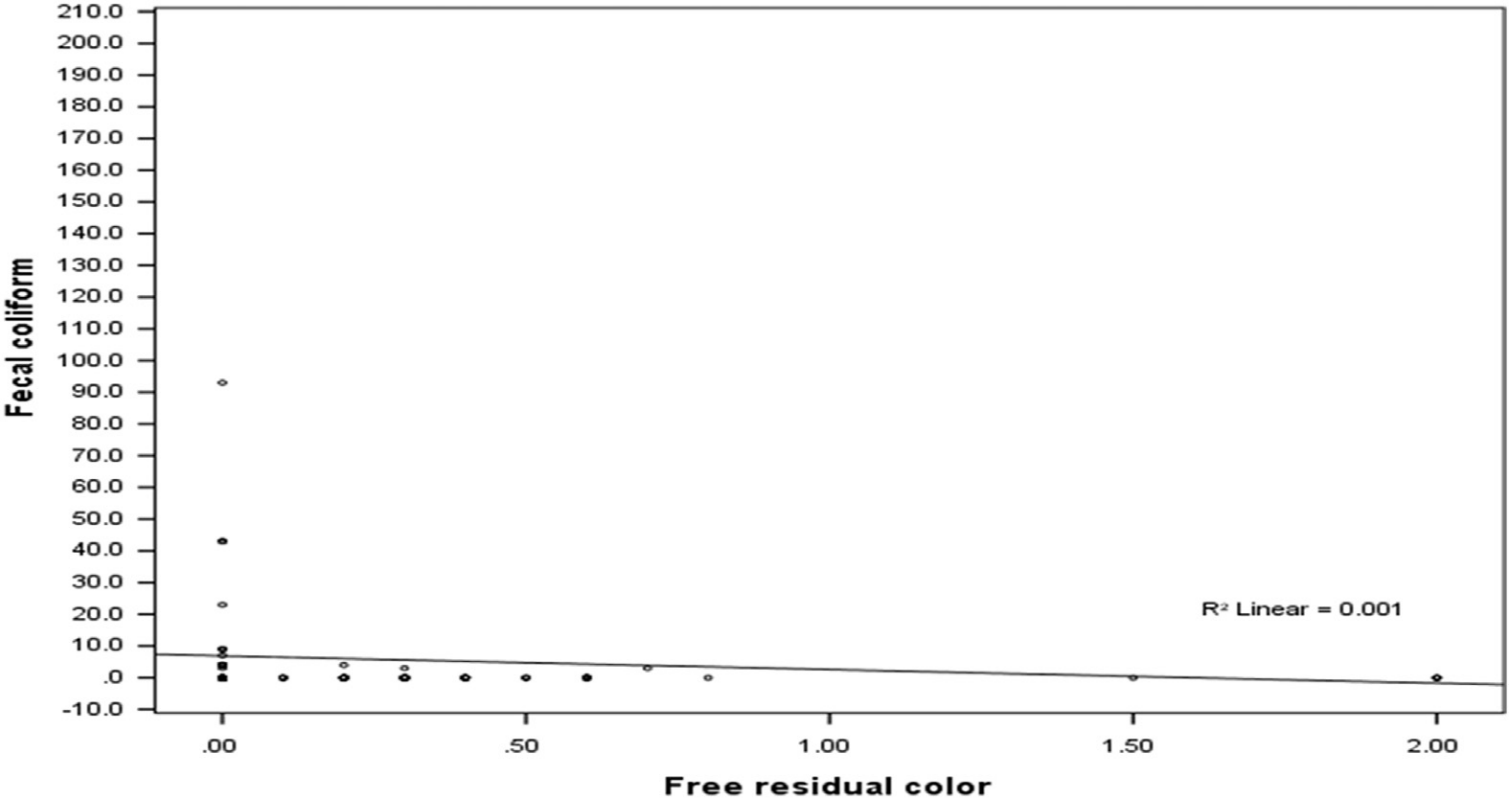
Relationship between Free chloration residual and fecal coliform in the different months in the drinking water of Poldasht's villages.

The mean, median, range and standard deviation of measured parameters in villages of Poldasht city have shown in Table 1, Table 2. The median range of pH was 7.53 ± 0.12. The amount of residual chlorine of samples was in the range of 0.12 ± 0.2 to 0.44 ± 0.53 mg/L. The mean coliform and fecal coliform were 15.54 ± 54.70 and 6.06 ± 40.69 vMPN/100 mL respectively. The maximum value of turbidity was 0.67 ± 0.56 NTU. In 99.5% of samples turbidity was lower than 5 NTU. There was a linear relation between the free residual color and Coliform in different month of follow up (*r* = −0.154, *P* < 0.001) (Table 3) (Fig. 1, Fig. 2, Fig. 3).

**Table 1:** Mean, median, range and standard deviation of measured chemical parameters in villages of Poldasht city.

**Month**	**Free residual color**		**pH**		**Turbidity**	
	Mean ± SD	Median (range)	Mean ± SD	Median (range)	Mean ± SD	Median (range)
September	0.25 ± 0.28	0.2 (0 to 1)	7.6 ± 0.1	7.6 (7.4 to 7.6)	0.31 ± 0.13	0.29 (0.12 to 0.54)
October	0.12 ± 0.2	0 (0 to 0.8)	7.6 ± 0.1	7.6 (7.4 to 7.6)	0.27 ± 0.07	0.26 (0.1 to 0.42)
November	0.28 ± 0.27	0.3 (0 to 1)	7.5 ± 0.1	7.6 (7.4 to 7.6)	0.55 ± 0.69	0.34 (0.12 to 3.5)
December	0.2 ± 0.27	0 (0 to 1)	7.5 ± 0.1	7.5 (7.4 to 7.6)	0.32 ± 0.06	0.32 (0.1 to 0.41)
January	0.44 ± 0.53	0.3 (0 to 2)	7.5 ± 0.1	7.6 (7.4 to 7.8)	0.45 ± 0.03	0.46 (0.4 to 0.49)
February	0.21 ± 0.34	0 (0 to 2)	7.6 ± 0.1	7.6 (7.4 to 7.8)	0.67 ± 0.56	0.5 (0.35 to 2.64)
March	0.34 ± 0.37	0.3 (0 to 1.5)	7.6 ± 0.1	7.6 (7.4 to 7.8)	0.45 ± 0.13	0.41 (0.21 to 0.66)
April	0.21 ± 0.4	0 (0 to 2)	7.5 ± 0.1	7.6 (7.4 to 7.6)	0.77 ± 0.58	0.61 (0.31 to 2.45)
May	0.34 ± 0.52	0.2 (0 to 2)	7.5 ± 0.1	7.6 (7.2 to 7.8)	0.46 ± 0.29	0.36 (0.19 to 1.76)
june	0.41 ± 0.53	0.3 (0 to 2)	7.5 ± 0.2	7.5 (7 to 8.2)	0.51 ± 0.08	0.49 (0.39 to 0.68)
July	0.22 ± 0.26	0.2 (0 to 0.8)	7.6 ± 0.1	7.6 (7.4 to 7.8)	0.4 ± 0.05	0.39 (0.31 to 0.54)
August	0.42 ± 0.57	0.2 (0 to 2)	7.5 ± 0.2	7.6 (7 to 7.6)	0.42 ± 0.04	0.43 (0.35 to 0.48)
P-value†	0.005		0.021		<0.001	

† Based on Linear Mixed model.

**Table 2:** Mean median range and standard deviation of measured microbial parameters in villages of Poldasht city.

**Month**	**Coliform**		**Fecal coliform**	
	Mean ± SD	Median (range)	Mean ± SD	Median (range)
September	18.6 ± 54.9	0 (0 to 240)	16.6 ± 55.2	0 (0 to 240)
October	17 ± 65	0 (0 to 460)	6.9 ± 24.8	0 (0 to 93)
November	18.7 ± 56.8	0 (0 to 240)	0.2 ± 0.7	0 (0 to 3)
December	2.7 ± 8	0 (0 to 43)	0 ± 0	0 (0 to 0)
January	6.3 ± 20.8	0 (0 to 93)	0 ± 0	0 (0 to 0)
February	2.2 ± 5	0 (0 to 23)	0.8 ± 2.7	0 (0 to 9)
March	3.6 ± 8	0 (0 to 43)	0 ± 0	0 (0 to 0)
April	6.9 ± 19	0 (0 to 93)	1.2 ± 1.9	0 (0 to 4)
May	21.7 ± 71.3	0 (0 to 460)	20 ± 93.7	0 (0 to 460)
June	21.8 ± 79.8	0 (0 to 460)	0.5 ± 1.6	0 (0 to 7)
July	44.2 ± 85.2	0 (0 to 240)	6.4 ± 15.5	0 (0 to 43)
August	15.8 ± 49.5	0 (0 to 240)	1.1 ± 2	0 (0 to 4)
P-value†	0.005		–	

† Based on Linear Mixed model.

**Table 3:** Correlation of residual chlorine with other parameter in rural areas of Poldasht basis on Linear Mixed Model.

		**pH**	**Coliform**	**Fecal coliform**	**Turbidity**
**Free**	**Pearson**	−0.058	−0.154**	−0.038	0.043
**residual color**					
	**Sig. (2-tailed)**	0.176	<0.001	0.624	0.311


** Correlation is significant at the 0.01 level (2-tailed).

## 2 Experimental design, materials and methods

### 2.1 Study area description

**Fig. 4:**
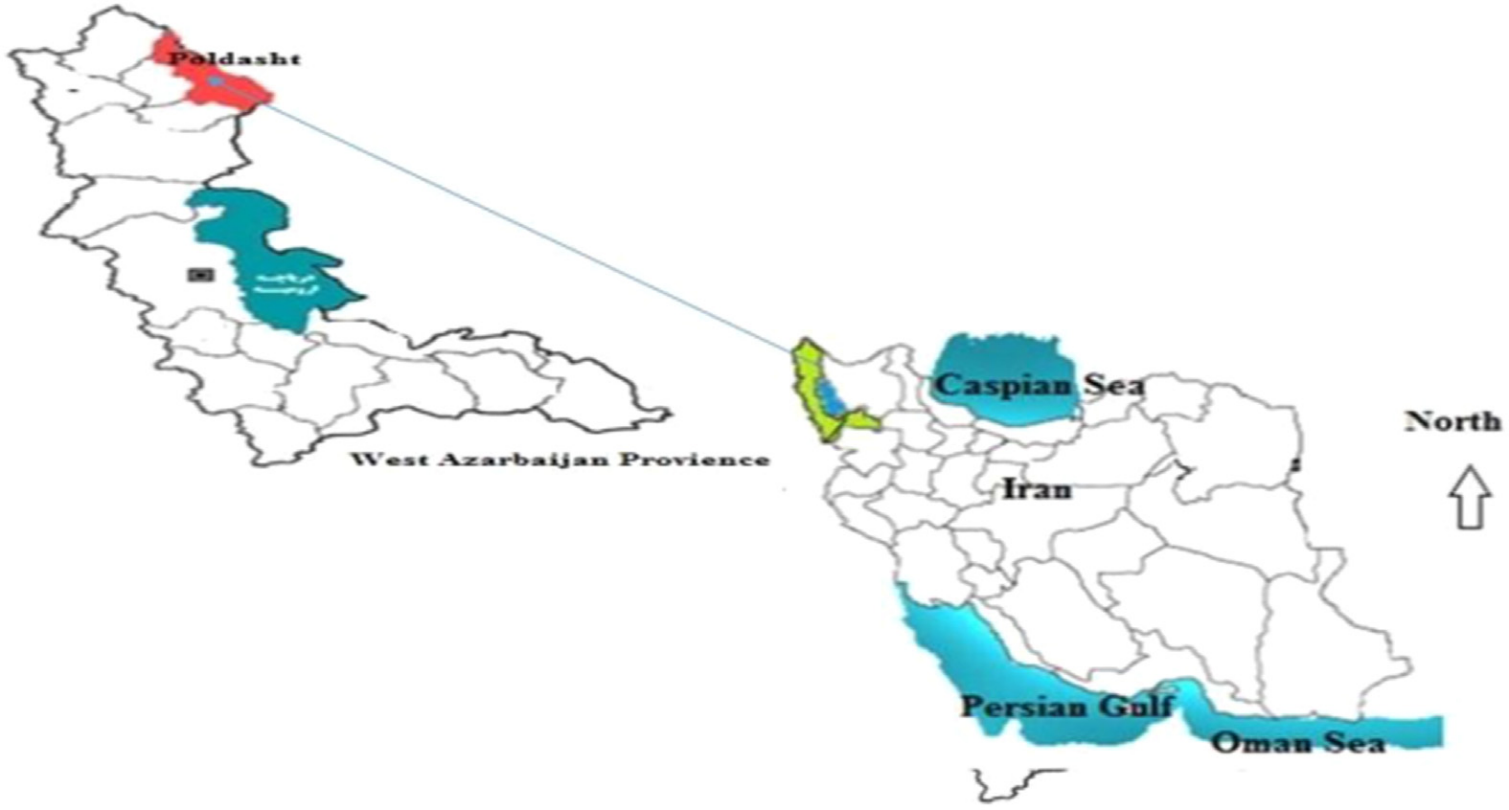
Location of the study area in Poldasht city, West Azerbaijan, Iran.

Poldasht county is located in North West Azerbaijan province of Iran and North Western with coordinates (UTM) *X* = 446,625 to 513,055 to the east and *Y* = 4,344,280 to 4,402,863 is located north latitude. Poldasht meteorological station showed that in a Long-term, the average rainfall was equal to 131.5 mm [1]. The city has also borderline from West and North with Turkey country (Fig. 4).

### 2.2 Sample collection and analytical procedures

This descriptive-analytic study was conducted on main drinking water supplies in 57 villages were selected in Poldasht rural county, west Azerbaijan Province, Iran. The samples comprised all villages equipped with active drinking water distribution system. 648 samples were taken from 57 villages during 12 month period to test for microbial quality according to the latest guidelines of WHO. Fecal coliform, coliform, Turbidity, pH and free residual chlorine were analyzed.

#### 2.2.1 Chemical properties

At the site, 250 ml polypropylene bottle containing 0.4 ml of a 10% sodium thiosulfate solution was used for collecting water samples and transported to laboratory in 6 h and 4 °C. Before the sampling, the sampling bottles were sterilized in autoclave apparatus. In order to determine the quality of water, the turbidity, free residual chlorine and pH were analyzed in the central water laboratory of Poldasht city. Free residual chlorine and pH were measured by a colorimeter kit on an annually basis. And also Turbidity test was done using HACH turbidity meter [2], [3], [4], [5].

#### 2.2.2 Microbial properties tests

Tests for indicator bacteria were done for the assessment of microbial properties of water samples. Indirect evidence of presence of pathogens was also confirmed by coliform test. Most probable number (MPN), confirmed test for coliform and total viable count or standard plate count were studied for microbial properties analysis.

#### 2.2.3 Most probable number (MPN)

A total of 648 water samples were taken from 57 villages during 12 month period to test for microbial quality in 2014–2015. Samples were collected in 250 ml sterile flasks. They were kept in ice boxes and sent to the microbiology laboratory for bacteriological examination. In the MPN method, a presumptive test was performed first. A series of fermentation tubes that contain Lauryl tryptose broth (Merck Company) were inoculated with the water sample and incubated for 24 h at 35 °C. The fermentation tube contains an inverted tube to trap gases that are produced by the coliform bacteria. After 24–48 h, the fermentation tube was examined for gas production. If gas production is observed by the end of 48 h, the presumptive test is positive; coliform bacteria were present in the sample. A confirmed test was then performed to determine if fecal coliform bacteria are present. For the confirmed test, some of the content of the fermentation tube is transferred with a sterile loop to a fermentation tube containing Brilliant green lactose bile broth (Merck Company). The sample was incubated in a water bath at 44.5 °C for 24 h. Gas production in the fermentation tube after 24 h is considered a positive reaction, indicating fecal coliform. Based on which dilutions showed positive for coliform and/or fecal coliform, a table of most probable numbers was used to estimate the coliform content of the sample [2], [3], [6], [7], [8], [9], [10], [11].

### 2.3 Statistical method

To present data we used mean, standard deviation, median and range.to evaluate the linear relation of free residual chlorine and Coliform, Fecal coliform, Turbidity. We used Pearson correlation coefficient. To assess the changes of different parameters during different month of follow up in the same area when considering the probable correlation we used Linear Mixed Model (LMM). All statistical analysis performed by SPSS software (IBM Corp. Released 2013. IBM SPSS Statistics for Windows, Version 22.0. Armonk, NY: IBM Corp.). P-value less than 0.05 considered statistically significant.
