# Supplementary material for: Data on microbiological quality assessment of rural drinking water supplies in Poldasht county
Source: Data Brief. 2018 Feb 7;17:763–9. doi: 10.1016/j.dib.2018.02.003 (PMC5988409; doi:10.1016/j.dib.2018.02.003)
Supplement: Supplementary file 1 — Supplementary material [file mmc1.docx]

**Conflict of Interest**

The authors of this article declare that they have no conflict of interests.
